# Long short-term memory for a model-free estimation of macronutrient ion concentrations of root-zone in closed-loop soilless cultures

**DOI:** 10.1186/s13007-019-0443-7

**Published:** 2019-05-28

**Authors:** Taewon Moon, Tae In Ahn, Jung Eek Son

**Affiliations:** Department of Plant Science and Research Institute of Agriculture and Life Sciences, Seoul National University, 1 Gwanak-ro Gwanak-gu, Seoul, 08826 Korea

**Keywords:** Environmental factor, Hydroponics, Machine learning, Model-free estimation, Paprika

## Abstract

**Background:**

Root-zone environment is considered difficult to analyze, particularly in interpreting interactions between environment and plant. Closed-loop soilless cultures have been introduced to prevent environmental pollution, but difficulties in managing nutrients can cause nutrient imbalances with an adverse effect on crop growth. Recently, deep learning has been used to draw meaningful results from nonlinear data and long short-term memory (LSTM) is showing state-of-the-art results in analyzing time-series data. Therefore the macronutrient ion concentrations affected by accumulated environment conditions can be analyzed using LSTM.

**Results:**

The trained LSTM can estimate macronutrient ion concentrations in closed-loop soilless cultures using environmental and growth data. The average training accuracy of six macronutrients was R^2^ = 0.84 and the test accuracy was R^2^ = 0.67 with RMSE = 1.48 meq L^−1^. The used values of input interval and time step were 1 h and 168 (1 week), respectively. The accuracy was improved when the input interval became shorter, but not improved when the LSTM consisted of a multilayer structure. Regarding training methods, the LSTM improved the accuracy better than the non-LSTM. The trained LSTM showed relatively adequate accuracies and the interpolated ion concentrations showed variations similar to those seen during traditional cultivation.

**Conclusions:**

We could analyze the nutrient balance in the closed-loop soilless culture, the model showed potential in estimating the macronutrient ion concentrations using environmental and growth factors measured in greenhouses. Since the LSTM is a powerful and flexible tool used to interpret accumulative changes, it is easily applicable to various plant and cultivation conditions. In the future, this approach can be used to analyze interactions between plant physiology and root-zone environment.

## Background

Horticultural crops provide humans with nutritious food and are cultivated worldwide. Protected cultivation methods are commonly used to ensure high quality, high yield crops. One goal of protected cultivation is to produce crops at maximum levels using minimal amounts of energy and resources. To meet this goal, soilless cultures have been applied globally and have exhibited greater benefits compared to soil cultures in terms of improved crop yield and quality. To achieve maximum efficiency, it is important to analyze the environments with which the plants interact; although interactions between plants and aerial environments have frequently been studied [4, 26, 42], studies on root-zones are rare due to the complexity of the root-zone environment.

Closed-loop soilless cultures are constantly being evaluated for sustainable agricultural research. In the closed-loop condition, root-zone is a crucial environment because nutrient solutions are reused and plants uptake ions selectively [34]; therefore, an imbalance of nutrients can occur and result in the accumulation of specific nutrients [24, 33, 39]. Accumulation of specific ions can increase deviations in nutrient solutions, resulting in reduced crop yields. To that end, root-zone environments in closed-loop soilless cultures should be adequately evaluated.

Because the electrical conductivity (EC) of a solution is linearly related to the total equivalents of ions in the solution [14], nutrient solution concentrations are controlled by measuring EC in most existing closed-loop soilless cultures. However, because EC does not show each ion concentration, the ion balance in EC-based control systems cannot be estimated. For example, the nutrient solutions were empirically refreshed with growth stage in an EC-based control system, but the solutions were roughly controlled [24, 25]. To monitor individual ions in real time, several ion-selective electrodes have been introduced, but it require sampling and calibration processes [22]. Moreover, an automated system to sample, measure, and drain the nutrient solution has been developed, but this system exhibited a sampling blockage period due to a drainage problem [8]. These past studies indicated that many problems need to be solved in order to adequately control nutrient solutions in closed-loop soilless cultures.

Recently, deep learning has been used to draw meaningful interpretations from complicated nonlinear data [15, 18, 43], and also showed meaningful result in agriculture. Recently, deep learning approach has been used to estimate CO_2_ concentrations in greenhouses with acceptable levels of accuracy [31]. As part of deep learning, long short-term memory (LSTM) is used to analyze time-series data, such as voice recognition, video recognition and natural language processing. LSTM has shown state-of-the-art performance and higher accuracy than previous algorithms in many regions [11, 30], and also can be used to predict root-zone EC in closed-loop soilless cultures [32]. Ions of nutrient solutions are also influenced by the accumulation of time-series factors such as greenhouse environmental influence, water supply, water drainage, and plant growth. Therefore, the objective of this study was to estimate macronutrient ion concentrations in closed-loop soilless cultures with LSTM using greenhouse environmental data and plant growth data.

## Methods

### Greenhouse and cultivation conditions

**Fig. 1:**
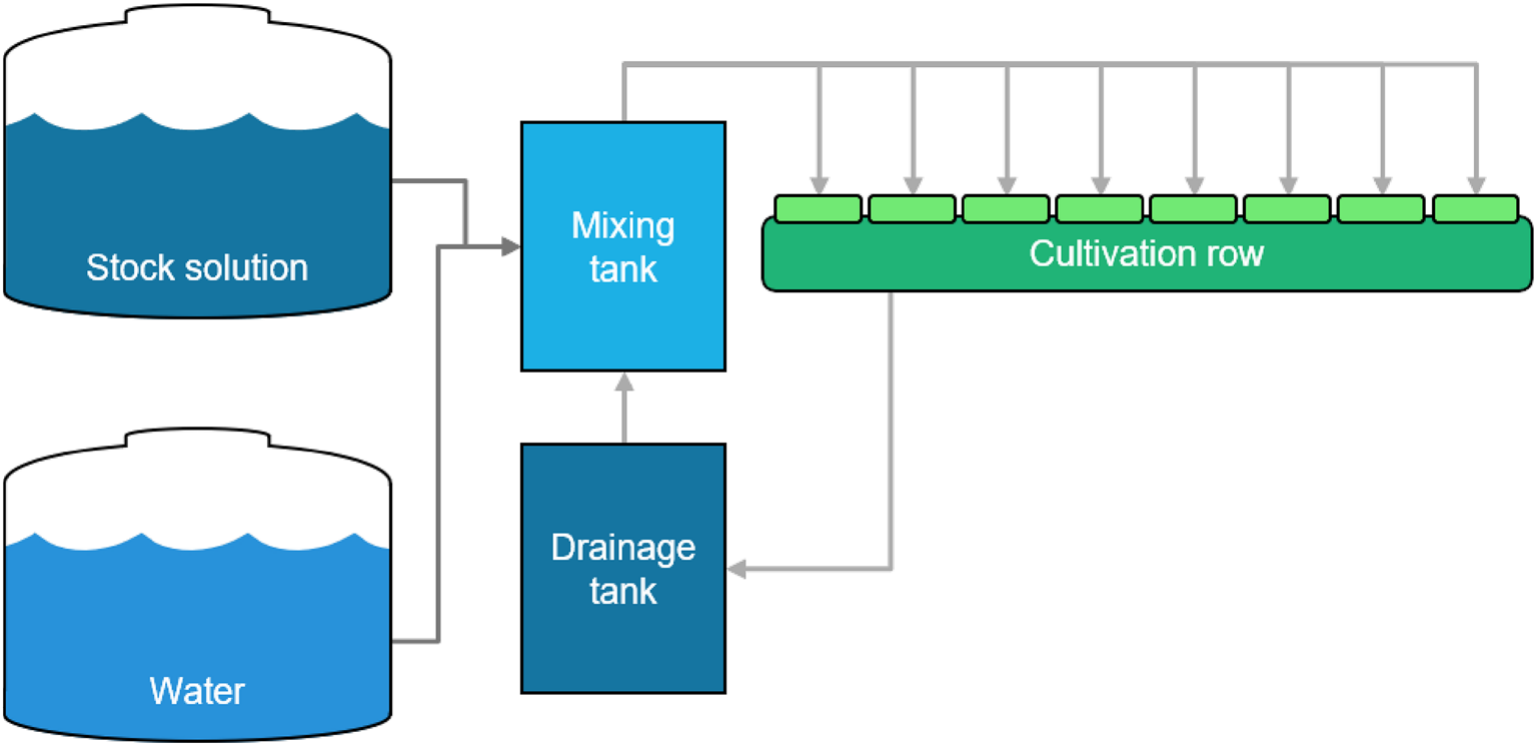
A diagram of the closed-loop cultivation system used in this study

**Fig. 2:**
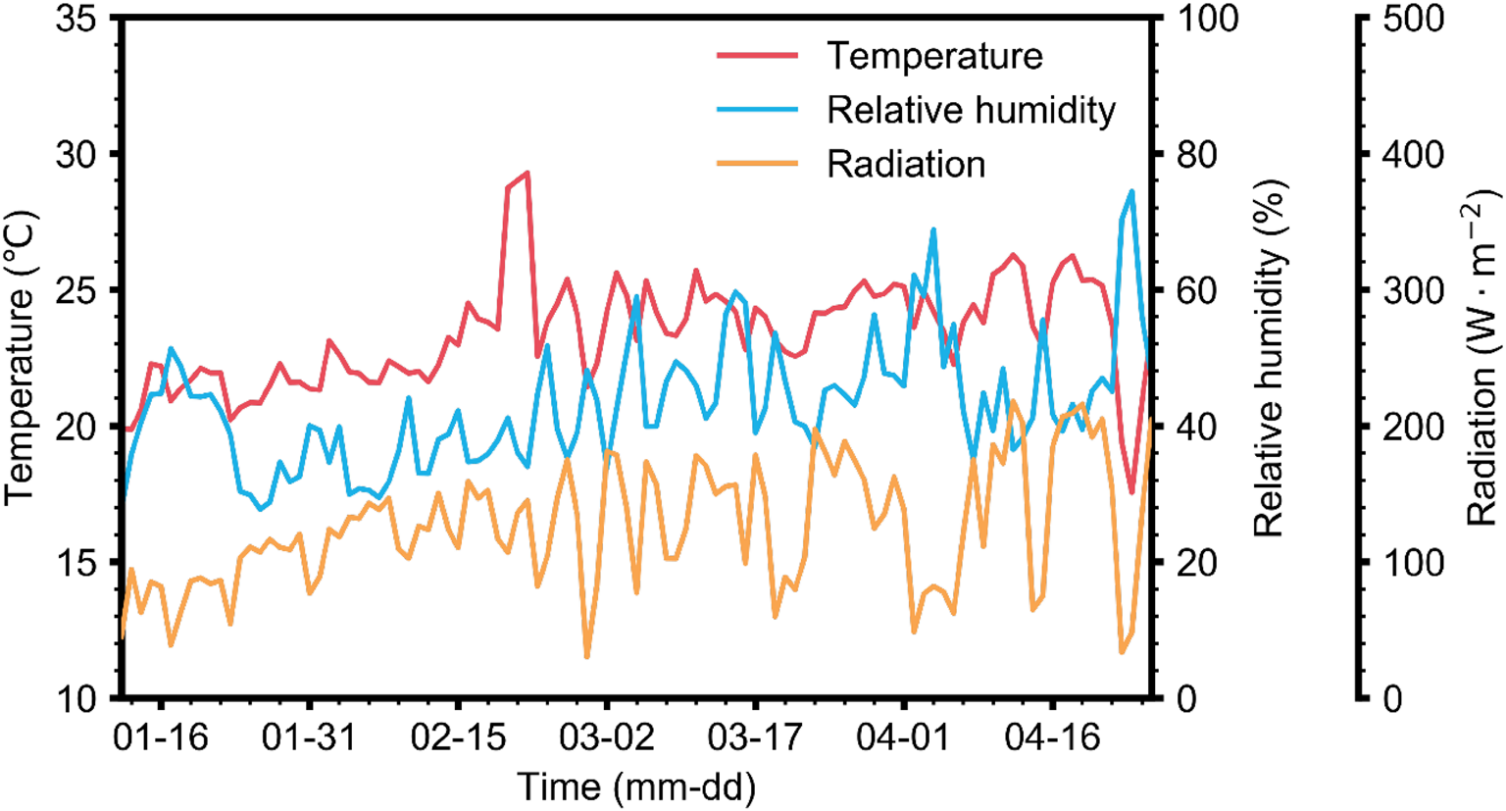
Daily average air temperature, relative humidity, and radiation measured in the greenhouse from January 12 to April 26, 2018

Data were collected from a Venlo-type greenhouse at the experimental farm of Seoul National University, Suwon, Korea (37.3°N 127.0°E). Four cultivation lines, each with seven rockwool slabs, were installed in the cultivation area. Five sweet pepper plants (*Capsicum annuum* L.) were grown per slab, and planting density was 5 plants m^−2^. Plants were sown on May 29, 2017 and transplanted on July 13, 2017. Each cultivation line had an independent closed-loop system with mixing tank, drainage tank and stock solution (Fig. 1). For this study, one line in the middle of the four cultivation lines was selected to maintain an average cultivation environment. Greenhouse daytime and nighttime temperatures were maintained at 25–30 °C and 17–22 °C, respectively (Fig. 2).

The composition of the stock solution was 14.17 meq L^−1^ of NO_3_^−^, 1.14 meq L^−1^ of H_2_PO_4_^−^, 5.92 meq L^−1^ of K^+^, 8.85 meq L^−1^ of Ca^2+^, 3.17 meq L^−1^ of Mg^2+^ and 3.20 meq L^−1^ of SO_4_^2−^ as macroelements; and 0.038 meq L^−1^ of Fe^2+^, 0.020 meq L^−1^ of Zn^2+^, 0.003 meq L^−1^ of Cu^2+^, 0.021 meq L^−1^ of Mn^2+^ and 0.001 meq L^−1^ of MoO_4_^2−^ as microelements. Nutrients were divided into two solutions, A and B, and the composition was based on the PBG nutrient solution of the Netherlands. EC of nutrient solutions was maintained between 2.6 and 4.0 dS m^−1^ and pH was maintained between 4.5 and 6.5. An integrated solar radiation method was applied for irrigation control. In this closed-loop system, water and stock solution were combined in a mixing tank prior to being used for irrigation, and any drainage was returned to the mixing tank (52 cm × 26 cm × 26 cm). EC and pH in the water tank was monitored every 3 days using a multimeter (Multi 3420 SET C, Wissenschaftlich-Technische-Werkstätten, Weilheim, Germany). EC and water content in root media were measured using a TDR sensor (WCM-control, Grodan, Roermond, the Netherlands). Fresh water mixed with the stock solution had EC and pH values of 0.17 dS m^−1^ and 7.11, respectively, and contained 0.21 meq L^−1^ of Na^+^, 0.29 meq L^−1^ of Cl^−^, 0.04 meq L^−1^ of K^+^, 0.71 meq L^−1^ of Ca^2+^, 0.21 meq L^−1^ of Mg^2+^, 0.19 meq L^−1^ of SO_4_^2−^, 0.39 meq L^−1^ of NO_3_^−^ and 0.04 meq L^−1^ of PO_4_^3−^. Drainage ratios were maintained at 50–60% during the experimental period. Plants were grown to maintain two main stems, which were vertically trellised to a “V” canopy system [20]. Data were collected 184–288 days after transplanting.

### Long short-term memory (LSTM)

**Fig. 3:**
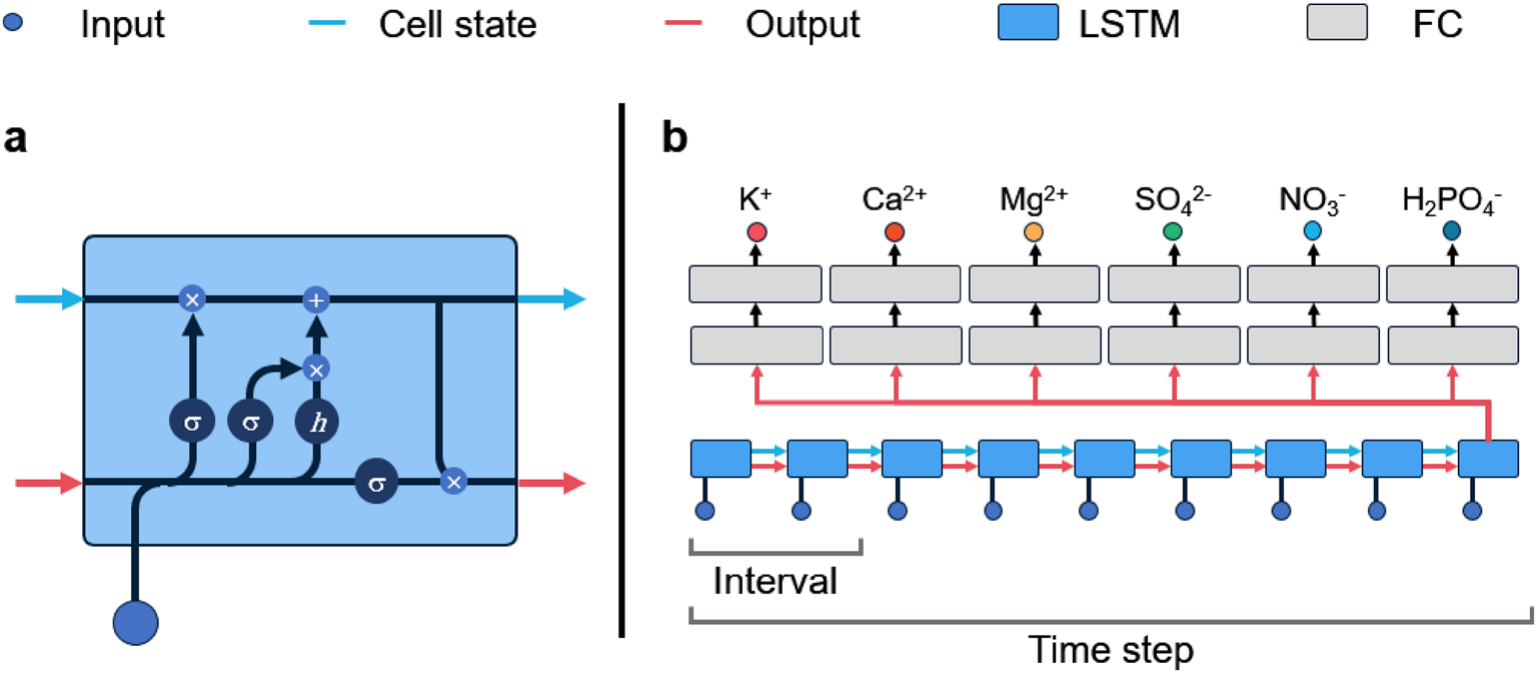
A structure of long short-term memory (LSTM, **a**) and a diagram of model training with multi-task learning (MTL, **b**). The abbreviation FC represents fully-connected layers, and the symbol *h* and *σ* represent the hidden layers with hyperbolic tangent and sigmoid as an activation function, respectively

LSTM has been used to analyze long sequential periods [16]. In this study, we used the many-to-one structure of LSTM (Fig. 3). Symbols *h* and *σ* represent a hyperbolic tangent function and a sigmoid function, respectively. LSTM not only multiplies but also adds sequence information, which solves problems associated with a vanishing or exploding gradient. LSTM accepts current input and previous output at the same time, and accepted values are operated at the gates. Information is saved in the cell state, so sequences can be processed by model training. Gates of LSTM are divided into three parts: the input gate determines input and output selections, the forget gate determines how much previous information should be forgotten, and the output gate mixes the cell state with input data. Many-to-one LSTM yields the final output when the computation step reaches the predetermined time step. In this study, multi-task learning (MTL) was applied [36]. The LSTM was shared by each task that predicted each ion, and two fully-connected layers yielded each ion concentration (Fig. 3b).

LSTM has hidden layers like ordinary artificial neural networks [10, 17]. Empirically, input and output activation functions were set to the hyperbolic tangent function, and the gate activation function was set to the sigmoid function. The number of neurons was varied to find optimal values. Stacking hidden layers does not change the accuracy significantly, so one hidden layer was used for LSTM [41, 44]. AdamOptimizer was used to train the LSTM [23]. The hyperparameters for the LSTM and AdamOptimizer were set to universal values except for learning rate and epsilon. Learning rate and epsilon were empirically optimized for regression in this study. For regularization, layer normalization was used [3]. Neural networks are trained to minimize cost [37], so mean square error (MSE) was used instead of root mean squared error (RMSE) as a cost-reducing computation. The coefficient of determination (R^2^) was used for training and testing accuracy, and RMSE was used to verify model robustness. Lastly, a multilayer perceptron (MLP) was used as a baseline model to verify accuracy by LSTM structure and MTL training. The MLP used had five hidden layers with 512 perceptrons. The inputs of MLP were the same as those of LSTM, but time step was not considered. TensorFlow (v. 1.9.0) was used for deep learning computations [1].

### Data collection and processing

Experimental data regarding nutrient solutions and growth environments were used for model training. EC and moisture contents were measured by EC sensors (SCF-01A, Dong Il Kyegi, Busan, Korea) and FDR sensors (CoCo 100B, Mirae Sensor, Seoul, Korea) (Table 1). Light intensity in the greenhouse was measured by a pyranometer (SP-110, Apogee, Logan, UT, USA). Data were measured every 10 s from January 12 to April 26, 2018. Because the 10-s interval was relatively short, mean values of specific intervals were used. Plant growth data were collected from five other plants on the same row every 3 days, and also mean data values were used for model training. In this study, fivefold cross validation was conducted. The ratio of test data were 20% in all validation processes.

**Table 1:** Data used as input for long short-term memory (LSTM) and their ranges

Input data (unit)	Range
Electrical conductivity (EC) of the substrate (dS m^−1^)	1.6–4.8
Moisture content of the substrate (%)	29.1–100.0
EC of nutrient solutions in the drainage tank (dS m^−1^)	0.0–6.0
Volume of nutrient solutions in the drainage tank (L)	0.0–11.3
Cumulative drainage volume per day (L)	0.0–182.2
Volume of nutrient solutions in the mixing tank (L)	1.7–14.1
Mixing volume of drainage (L)	0.0–4.9
Mixing volume of water (L)	0.0–7.5
Mixing volume of stock solution (L)	0.0–0.17
Cumulative irrigation volume per day (L)	0.0–196.0
Preset radiation integral for irrigation control (J cm^−2^)	0.0–150.0
Target volume of irrigation per dripper (mL)	160.0–220.0
Root-zone temperature (°C)	10.5–44.9
Root-zone pH	2.7–4.6
Light intensity (W m^−2^)	0.0–492.7
Greenhouse temperature (°C)	10.7–44.3
Greenhouse relative humidity (%)	8.6–83.9
SPAD	40.9–74.7
Plant height (cm)	115.0–217.0
Plant diameter (mm)	13.0–16.8
Number of nodes	16–40

Substrate nutrient solutions were sampled to determine ion concentrations. Sampling was conducted daily at 4:00 P.M. The concentrations of K^+^, Ca^2+^, Mg^2+^, SO_4_^2−^ and H_2_PO_4_^−^ were measured using an inductively-coupled plasma atomic emission spectrometer (VARIAN 730ES, Varian, Sydney, Australia). The concentration of NO_3_^−^ was measured using an ion chromatographer (ICS-3000, Dionex, Sunnyvale, CA, USA). Missing environmental data were also interpolated using linear interpolation, including missing values of EC and pH which were interpolated using manually-monitored data. Sixty-six points were used for the experiment; afterward, daily ion concentrations over the experimental period were estimated and analyzed using a trained LSTM.

To train the LSTM without biasing some features, data were normalized within the range of 0–1. Neural networks could not converge without normalization [3]. In this study, previously acquired data regarding nutrient solutions and growth environments were used as input, and time step of LSTM and interval of input data were varied to determine optimal values. The amount of input data was determined according to the time step and interval based on when the ions were sampled. Concentrations of K^+^, Ca^2+^, Mg^2+^, SO_4_^2−^, NO_3_^−^ and H_2_PO_4_^−^ were used as outputs (i.e., the number of tasks was six).

### Interpolation of unmeasured ion concentrations

**Fig. 4:**
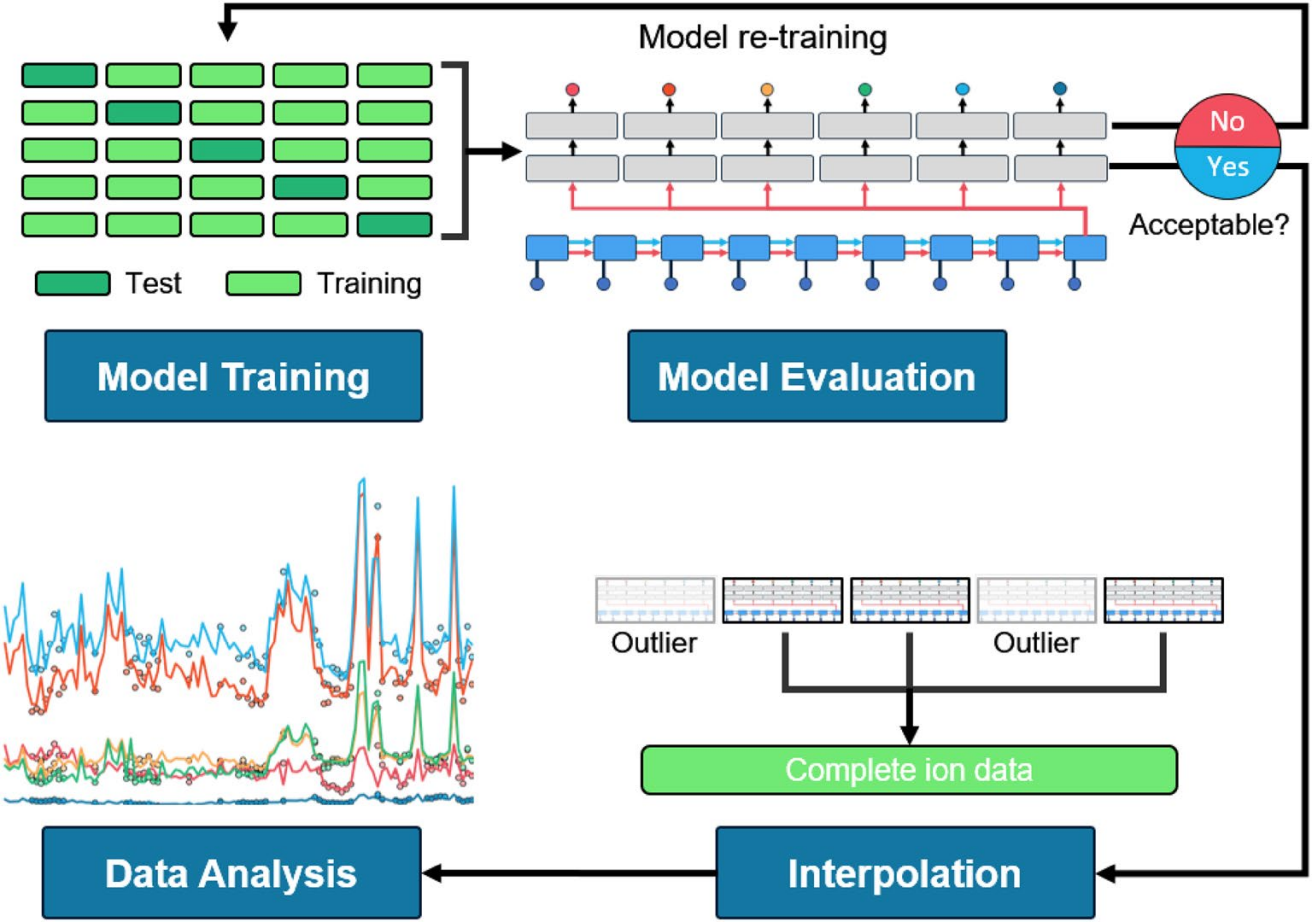
A methodology for interpolating unmeasured ion concentrations and analyzing changes in root-zone macronutrients

After the LSTM was trained and showed adequate accuracy, it was used to interpolate ion concentrations not measured (Fig. 4). The average of the five models from fivefold validation was used for the interpolating value. Maximum and minimum outlier models were excluded, so three values were averaged for the interpolation. In this study, plant growth data and environmental data were measured during the entire experimental period, while ion concentrations were measured over specific periods. In the model training, the LSTM was then trained and evaluated using the data measured during the specific periods. Finally, the trained LSTM interpolated ion concentrations over the entire experimental period and ion changes in the root-zone were analyzed.

## Results

### Plant growth and root-zone changes

**Fig. 5:**
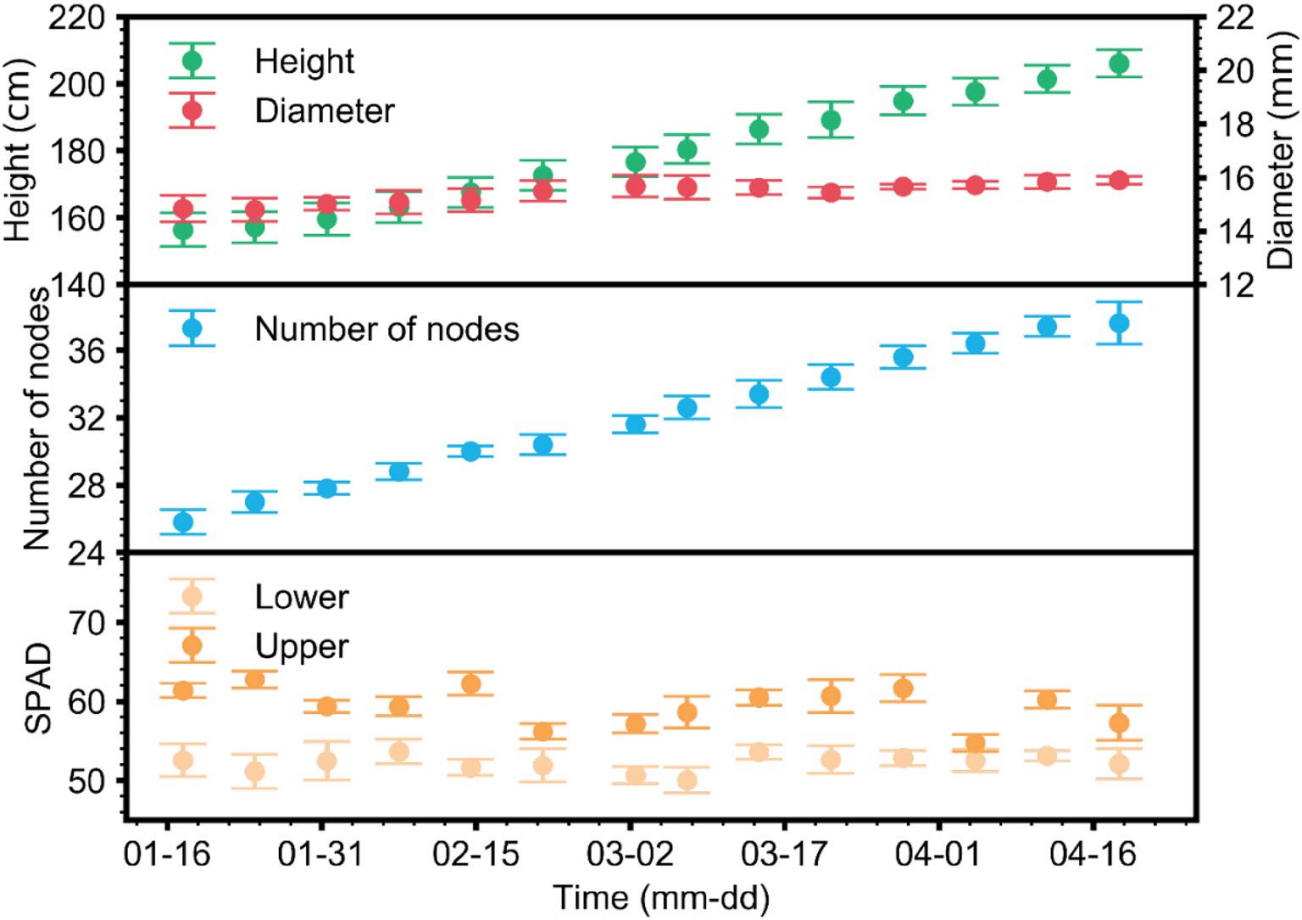
Plant height, diameter, number of nodes, SPAD of upper leaves and SPAD of lower leaves measured from January 12 to April 26, 2018. Average values of five plants from each line were used to train the LSTM

Plant growth was stable and normal throughout the experimental period (Fig. 5). Plant heights and the number of nodes showed sigmoidal patterns, while diameters showed no significant differences over time. The ranges of heights and diameters were 156–206 cm and 14.8–15.9 mm, respectively. The number of nodes changed at the range of 25–38 nodes. Soil plant analysis development (SPAD) values showed relatively constant values regardless of the period, but values for upper leaves were higher than for lower leaves.

**Fig. 6:**
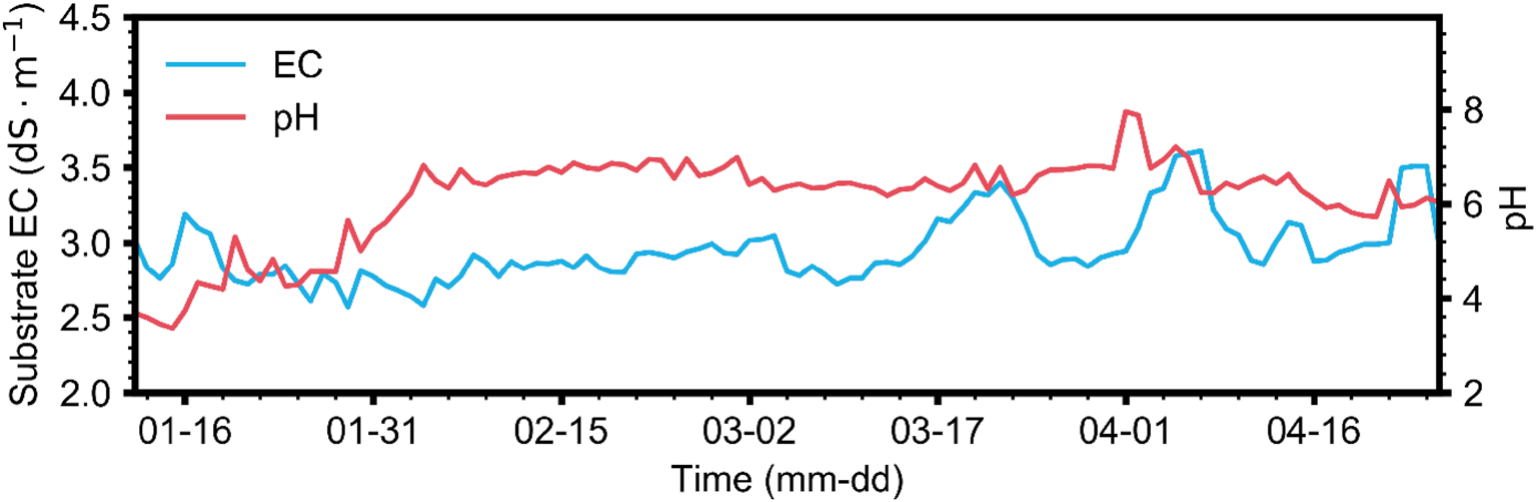
Daily average changes in substrate EC and pH from January 12 to April 26, 2018

The substrate EC and pH were disturbed during the last part of the experimental period (Fig. 6). EC fluctuated during the latter part of the cultivation, while pH increased before stabilizing between 6 and 7. EC and pH ranged from 2.6 to 3.6 dS m^−1^ and 3.4–8.0, respectively. Although pH fluctuated approximately 10% out of the control range, the nutrient solutions were managed within the normal range.

**Fig. 7:**
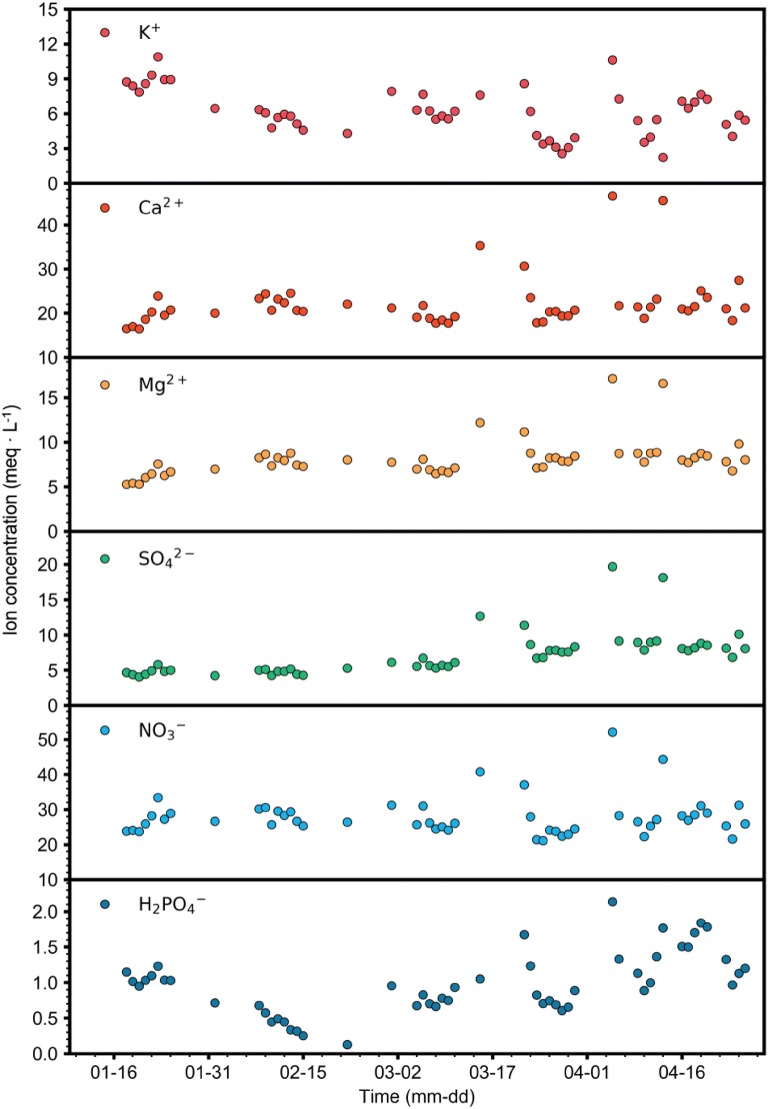
Target ion concentrations in the root-zone of a closed-loop soilless culture measured from January 12 to April 26, 2018

Ion concentrations also showed deviances (Fig. 7). Measured concentrations tended to be disturbed during the latter part of the growth stage. With the exception of outliers during the late growth stage, overall ion concentrations were generally constant. However, the concentrations of K^+^ slightly decreased over time, while Mg^2+^ and SO_4_^2−^ concentrations tended to increase. H_2_PO_4_^−^ fluctuated more than other ions, but the concentration range was lower.

### Validation of the trained LSTM

**Fig. 8:**
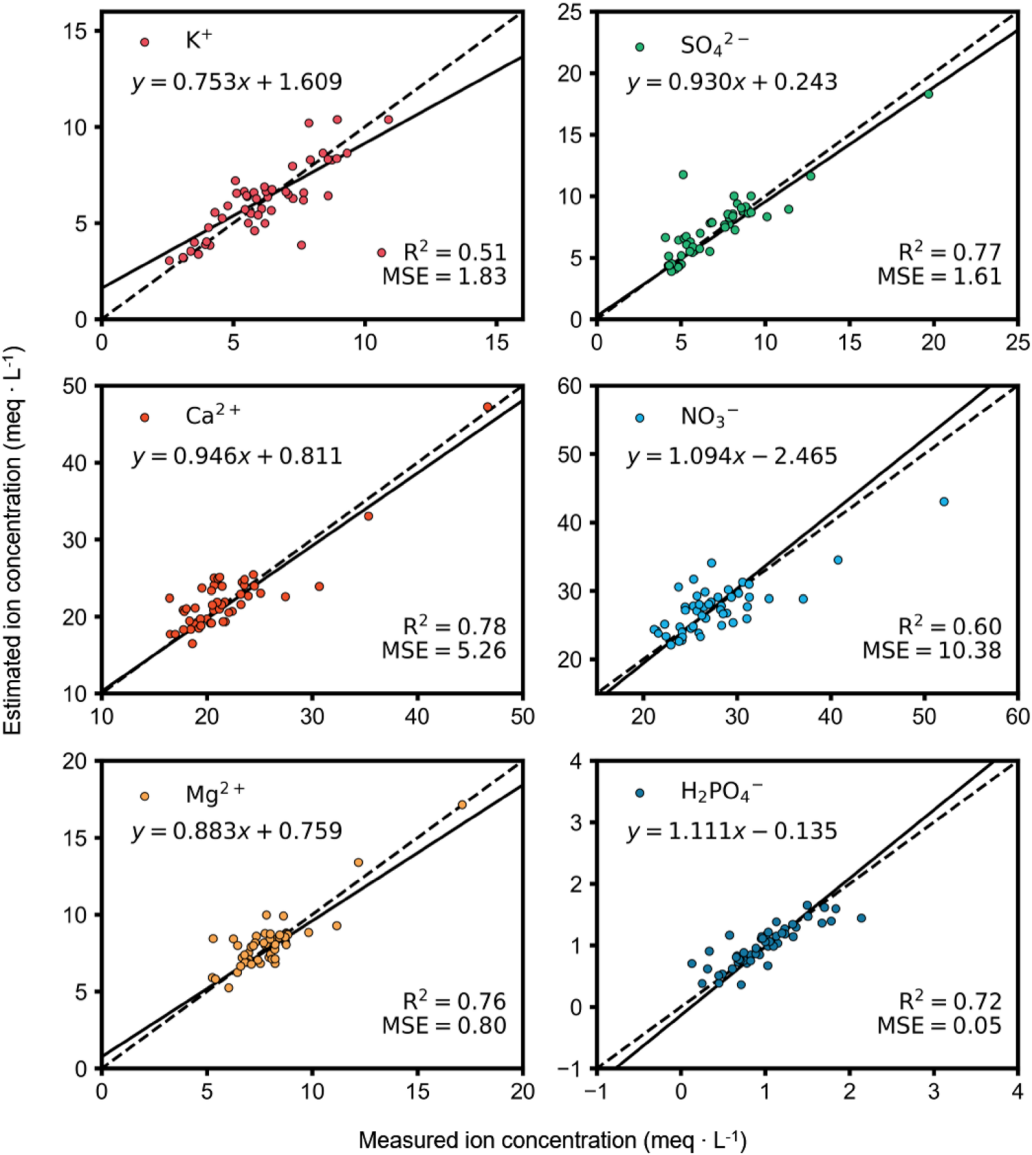
Comparison between measured and estimated ion concentrations under closed-loop cultivation conditions. All of the test data from cross validation were represented. The unit of RMSEs are meq L^−1^

As a result of experimentation with varied hyperparameters, the average test accuracy varied depending on the structure (Table 2). Even in the best combination, the test accuracy of each ion varied with ion concentration in the range of 0.51–0.78. Average accuracy of the best results was R^2^ = 0.67, RMSE = 1.48 meq L^−1^ (Fig. 8). Average accuracy of the training data was R^2^ = 0.84. Estimating the concentration of Ca^2+^ showed the highest accuracy, while the lowest accuracy was shown estimating the concentration of K^+^. In terms of the parameters, the learning rate almost did not affect the accuracy of the training. Optimal values of the input interval and the time step were 1 h and 168 (1 week), respectively. The trained model showed that accuracy improved when the interval became shorter. Regardless of the interval of input data, accuracy usually decreased when the time step was shorter than 1 week. Conversely, a time step longer than 1 week did not increase accuracy. In terms of the structure, accuracy was not improved when the LSTM consisted of a multilayer structure. Regarding training methods, the LSTM improved the accuracy better than MTL (Table 3). Using both improving methods resulted in the highest accuracy, and accuracies plummeted when the LSTM structure was not used.

**Table 2:** The average test accuracy of the six macronutrients (R^2^) for long short-term memory (LSTM) using a combination of hyperparameters

Number of perceptrons	Time step	Interval	Dropout probability	Learning rate	R^2^
8	168	1 h	1.0	0.001	0.58
64	168	1 h	1.0	0.02	0.64
128	1008	10 min	0.9	0.045	0.66
256	144	10 min	0.9	0.045	0.52
512	12	1 h	0.9	0.001	0.61
512	108	1 h	0.9	0.001	0.62
512	168	1 h	0.5	0.001	0.43
*512*	*168*	*1* *h*	*0.9*	*0.03*	*0.67*
512	168	1 h	0.9	0.001	0.66
512	168	1 h	1.0	0.03	0.65
512	336	30 min	0.9	0.03	0.44
512	336	1 h	0.9	0.03	0.66
1024	168	1 h	0.9	0.03	0.60

A combination of hyperparameters that showed the best accuracy is in italics

**Table 3:** Accuracy according to the existence of long short-term memory (LSTM) and multi-task learning (MTL)

Methodology	R^2^	RMSE (meq L^−1^)
With LSTM, with MTL	0.67	1.58
With LSTM, without MTL	0.51	1.66
Without LSTM, with MTL	− 0.75	3.90
Without LSTM, without MTL	− 0.54	3.62

R^2^ and RMSE represent the average values of the six ions

### Interpolating ion concentrations for all periods

**Fig. 9:**
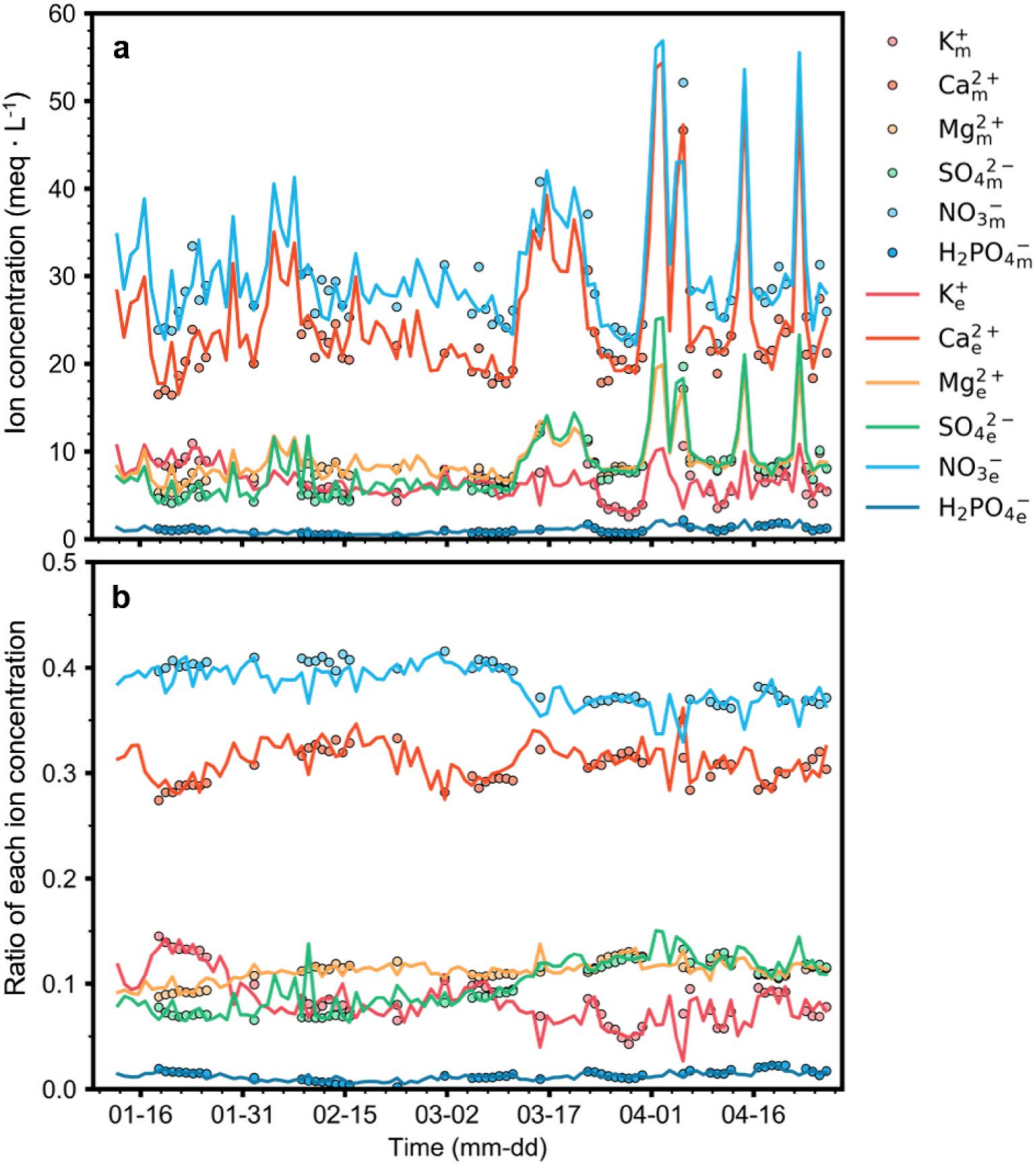
Comparisons of measured and interpolated ion concentrations using the trained long short-term memory (LSTM, **a**) and the ratio of each ion concentration to total ion concentration (**b**) from January 12 to April 26, 2018. The letters *m* and *e* represent measured and estimated values, respectively. All measured data were used for training or test in cross validation

Interpolation of ion concentrations was smooth at the beginning of the experiment, but large variations occurred later in the study (Fig. 9). NO_3_^−^ and Ca^2+^ showed relatively large deviances compared to other ions, and SO_4_^2−^ concentration was also highly variable. The ratio of K^+^ and NO_3_^−^ tended to decrease over time, while all other ions tended to increase. After mid-March, ion concentrations became highly variable and were relatively high, but decreased to normalized values within a week. Ion concentrations had high deviances, but the ratio of each ion concentration was relatively stable (Fig. 9b).

## Discussion

### The robustness of the trained multi-tasking LSTM

The accuracies of the ion concentrations varied per ion (Figs. 7, 8), which may be typical for nutrient solutions as previous studies have shown that variability in ion concentrations are dependent on each ion [8, 22]. Ion uptake in plants differs depending on the individual ions and varies with growth stage. Our results suggest that the LSTM was appropriately trained and was robust because it exhibited adequate accuracies for six different ions despite ionic differences. In an open condition, only the concentration of water and stock solution should be considered when replenishing the nutrient solution [29, 35]. However, restricted drainage in closed-loop conditions resulted in wider variation in ion concentrations, which made estimation of ion concentrations difficult and resulted in relatively low accuracies. Despite the wider variation in ion concentrations, the accuracies were still high enough to say the model was relatively robust.

In this study, learning rate increased because of normalization method [5]. However, the rate had little effect on the accuracy. It seemed that the highest accuracy is already yielded in relatively low learning rate (0.001), which used generally in neural network training. Although we did not compare all of the parameter combinations, converging speed of training seemed to be faster in higher learning rate, but the model unstably converged with high variance. In contrast, lower learning rate stably converged to the global minima, at least to the best model, but it needed more time to converge than higher learning rate. By the nature of the neural network training, once the LSTM converged, it can be used as an accurate model. Therefore, high learning rate is acceptable as long as the LSTM converge. After the training, model robustness was acquired using a 1-h interval and 168 time steps, so these settings were selected as the optimal condition although a time step of 1008 with a 10-min interval increased R^2^ by 0.01. Usually, irrigation disturbs ion concentrations, but irrigation time was often less than 1 h [40]. In this study, irrigation was determined by the integrated radiation, and the time of ion sampling was fixed at 4:00 P.M.; therefore, the time of the irrigation event would be different for each sampling time, but could not be considered due to the interval. That is, the data at the 1-h interval could reduce the sensitivity of the model. The model would have higher accuracy if the interval and time step were adjusted. Regardless of the intervals, we did not see improved accuracies in cases when the time step was longer than 1 week. The exact cause is unknown, but information that is longer than 1 week does not have a significant impact on the environment or plant changes.

Similarly, the structure of the LSTM did not significantly affect the accuracy. The dimension of the input data processed at one time is usually much larger than the dimension of the data used for this study when the LSTM is used [6, 7]. Because the dimension of the data in this study was about 20, the relationship between the ion concentrations and the plant environment may be found once a sufficient time step and interval were determined. Likewise, the number of layers did not affect accuracy because the data were simple enough that a multilayer was unnecessary.

As reported in other studies, accuracies improved when MTL was used for model training [19]. Previous studies have shown that increased model robustness was acquired by constructing a model to learn various tasks simultaneously (such as sentiment prediction and question type classification) using MTL in natural language processing [45]; therefore, accuracies could increase if EC, pH, or other greenhouse environmental factors are processed at the same time as the ion concentrations.

The accuracies using LSTM were significantly higher because of the large amount of information used to process time-series data [13, 21]. A LSTM structure can obtain information from input data by as much as the time step, while a non-LSTM structure cannot. If a non-LSTM structure attempted to use the same amount of input data as a LSTM, the structure would be overloaded and make computation inefficient. Estimating ion concentrations using MTL and the LSTM is the more appropriate method.

In addition, the LSTM is a model-free method, so application and modification of the model is relatively easy. Because most of the greenhouse environmental data is time-series data, it can be used to estimate plant growth if the same model is trained using different data. The trained LSTM can also be applied to different domains or conditions using relatively small amounts of data if transfer learning is used [12], i.e., the trained LSTM could be applied to estimate ion concentrations under different conditions such as other greenhouses, plant factories, or crops.

### Reasonability of interpolated ion concentrations

The deviance of both the ions and actual measured ion concentrations was large (Fig. 9a), but the ratio of the ions changed in reasonable range (Fig. 9b). These results suggest that the interpolation of the ion concentrations was a reasonable approach, and the deviance was due to the nature of the closed-loop condition. One characteristic of a closed-loop condition is frequent variation in ion concentrations toward the latter stages of cultivation [2, 24]. After April, ion concentrations greatly increased before returning to prior levels, but the ion concentrations fluctuated similar to the increasing tendency of the EC (Fig. 6). Therefore, the trained LSTM had inferred the relationship between EC and ion concentrations appropriately. Total ion concentrations are known to be positively correlated with EC and pH [38], similar to the changes of known ion concentrations, so the interpolated ion concentrations were reasonable.

### Analysis of ion uptake in plant

Nutrient uptake of sweet peppers is largely unchanged after completion of growth [27]. During the latter period of cultivation, fruit removal can affect nutrient uptake [28]. However, in this study ion concentrations sharply changed, so the effect of fertilization was likely to be greater than the disturbance due to the nutrient uptake.

NO_3_^−^ and Ca^2+^ showed relatively large deviances compared to other ions. Sweet pepper plants have a high uptake of NO_3_^−^ and Ca^2+^, so the amount of NO_3_^−^ and Ca^2+^ in nutrient supplies are large [9, 27]. We saw no rapid change in EC and pH, so other unmeasured ions accumulated during the latter part of cultivation could disturb the root-zone nutrients. Therefore, nutrients were supplied in relatively high doses so that NO_3_^−^ and Ca^2+^ would be the most affected ions based on their abundance.

Sweet pepper plants also have high uptake of K^+^ [27]. If the renewal period of the nutrient solution is not appropriate, the amount of K^+^ and NO_3_^−^ can decrease [2, 25]. In this study, the ratio of these ions decreased at a very small rate indicating that the renewal interval was close to the optimal value; however, fine tuning of the interval time is possible according to the interpolated ion concentrations. Therefore, the interpolation of ion concentrations could also help optimize the renewal interval of nutrient solutions.

## Conclusions

The model trained in this study estimated ion concentrations in soilless cultures, and the LSTM was used as a deep learning approach. After model training, accuracy R^2^ values ranged between 0.51 and 0.78 for six macro ions. The trained LSTM could interpolate daily ion concentrations, and ion concentrations were within reason. The interpolated ion concentrations showed variation similar to those seen during traditional cultivation. However, the model accuracy is limited in this study, so the same accuracy cannot be guaranteed in changed conditions. The trained model could be applied to differing cultivation conditions or domains such as plant factories, other pepper varieties or other crops. Neither Na^+^ nor Cl^−^ were considered in this study, but studies on other ions could be conducted using these same methods. Because the LSTM can be used to analyze accumulative changes, further studies predicting future ion concentrations based on predicted environment changes could be conducted. Stable and continuous measurement systems could improve the model robustness. This methodology can be used to interpret the interaction between the root-zone environment and plants in future research.

## Data Availability

All data generated or analysed during this study are included in this published article.
